# A Protective Role of Glibenclamide in Inflammation-Associated Injury

**DOI:** 10.1155/2017/3578702

**Published:** 2017-06-27

**Authors:** Gensheng Zhang, Xiuhui Lin, Shufang Zhang, Huiqing Xiu, Chuli Pan, Wei Cui

**Affiliations:** ^1^Department of Critical Care Medicine, Second Affiliated Hospital, Zhejiang University School of Medicine, Hangzhou, Zhejiang 310009, China; ^2^Department of Cardiology, Second Affiliated Hospital, Zhejiang University School of Medicine, Hangzhou, Zhejiang 310009, China

## Abstract

Glibenclamide is the most widely used sulfonylurea drug for the treatment of type 2 diabetes mellitus (DM). Recent studies have suggested that glibenclamide reduced adverse neuroinflammation and improved behavioral outcomes following central nervous system (CNS) injury. We reviewed glibenclamide's anti-inflammatory effects: abundant evidences have shown that glibenclamide exerted an anti-inflammatory effect in respiratory, digestive, urological, cardiological, and CNS diseases, as well as in ischemia-reperfusion injury. Glibenclamide might block K_ATP_ channel, Sur1-Trpm4 channel, and NOD-like receptor pyrin domain containing 3 (NLRP3) inflammasome activation, decrease the production of proinflammatory mediators (TNF-*α*, IL-1*β*, and reactive oxygen species), and suppress the accumulation of inflammatory cells. Glibenclamide's anti-inflammation warrants further investigation.

## 1. Introduction

Glibenclamide, an oral hypoglycemic agent, belongs to the class of sulfonylureas, and its clinical utilization dates back to the 1960s [1]. Actually, sulfonylureas were discovered accidentally, as the antimicrobial sulfonamides caused hypoglycemia in animals. Thereafter, glibenclamide has been used widely in the type II diabetes mellitus (DM).

The mechanism of glibenclamide in DM treatment is due to its inhibition of ATP-sensitive potassium channel (K_ATP_) (Sur1-Kir6.2) in pancreatic *β* islet cells, which leads to the depolarization of *β* cell plasma membrane and activation of voltage-gated calcium channels. Calcium influx triggers insulin release from *β* cells [2, 3]. Glibenclamide is an K_ATP_ channel blocker and broad-spectrum ATP-binding cassette transporter (ABC) inhibitor. K_ATP_ channels are widely distributed in muscle, pancreatic beta cells, and the brain. Their activity is regulated by adenine nucleotides, activated by falling ATP and rising ADP [4]. K_ATP_ channel is a heterooctamer consisting of four pore-forming subunits (Kir6.x) and four regulatory sulfonylurea receptor (Sur) subunits [5] composed in a 1 : 1 stoichiometry as a tetramer (Sur-Kir6.X)_4_. This complex conducts potassium ions across cell membranes and thereby couples cellular energy metabolism to membrane electrical activity. Three isoforms of Sur (Sur1, Sur2A, and Sur2B) have been cloned and are specified as pancreatic, cardiac, and (vascular) smooth muscle types, respectively [6]. The Kir subunit is either Kir6.1 or Kir6.2 [6]. Different combinations of Kir6.X and Sur isoforms in different cellular/tissue distributions result in functional diversity [5]: the classical *β* cell type and neuronal type with the combination of Kir6.2 and Sur1 (SUR_1_-Kir6._2_)_4_; the cardiac and skeletal type with the combination of Kir6.2 and Sur2A (Sur2A-Kir6.2)_4_; the smooth muscle type with the combination of Kir6.2 and Sur2B (Sur2B-Kir6.2)_4_; or Kir6.1 and Sur2B (Sur2B-Kir6.1)_4_ [5].

Besides its hypoglycemia effects, glibenclamide recently has been shown to play role in inflammation regulation. In addition to combining with the Kir6.X subunit to form the K_ATP_ channel, Sur1 also associates with an ATP- and calcium-sensitive nonselective cation channel to form Sur1-Trpm4 channels (previously called the Sur1-NCCa-ATP channel). Interestingly, but not surprisingly, glibenclamide has been found to inhibit Sur1-Trpm4 channels by directly binding the Sur1 subunit to protect against inflammation-associated injury in the central nervous system (CNS) [7]. Activation of Sur1-Trpm4 channels depolarizes the cell membrane, which is associated with cell death and cerebral edema [8]. Glibenclamide reduces adverse neuroinflammation and behavioral outcomes in CNS injury [9]. Furthermore, glibenclamide displays a protective role in inflammation-induced injury in various systems, including respiration [10–12], digestion [13], urology [14–16], cardiology [17], CNS [18–21], some special conditions such as melioidosis [12, 22] and ischemia-reperfusion (IR) injury [23–26], and septic shock [27–29]. Here, we review anti-inflammatory effects of glibenclamide and its possible mechanisms.

## 2. Anti-Inflammatory Roles of Glibenclamide

### 2.1. Glibenclamide and Respiratory Diseases

K_ATP_ channels are expressed on the basolateral membrane of airway epithelial cells. K_ATP_ channel activation stimulates proliferation of fibroblasts, hepatocytes, and epithelial cells and induces migration of neutrophils and airway epithelial cells [30–32].

Bronchopulmonary dysplasia is a devastating lung complication in preterm infants. Inflammation plays a critical role in bronchopulmonary dysplasia development [33–35]. Recently, Liao et al. found that glibenclamide (5 *μ*M) protected neonatal mice from developing bronchopulmonary dysplasia, which was associated with inhibition of caspase-1 activation, reduction in interleukin-1 beta (IL-1*β*) production, and suppression of neutrophils and macrophages influx [10].

Allergic asthma is characterized by excessive T-helper type 2 (Th2) immune response, large eosinophilic airway inflammation, airway hyperresponsiveness (AHR), and mucus hyperproduction, and subsequent airway remodeling [36]. In a mouse model of ovalbumin-induced asthma, glibenclamide significantly reduced eosinophils in bronchoalveolar lavage fluid and reduced Th2-associated cytokines like IL-5 and IL-13, therefore attenuated airway inflammation and AHR [11]. In vitro, glibenclamide also significantly downregulated ovalbumin-stimulated Th2 cytokine release, such as IL-5, IL-4, and IL-13 [11]. The authors attributed this anti-inflammatory effect to inhibition of IL-4/IL-13/p-STAT6/VCAM-1 signaling pathway.

Acute lung injury/acute respiratory distress syndrome (ALI/ARDS) is also associated with an overwhelming inflammatory response: proinflammatory cytokines IL-1*β* and IL-18 levels are high in bronchoalveolar lavage fluid from patients with ALI/ARDS and correlate with mortality [37]. Melioidosis patients pretreated with glibenclamide showed lower accumulation of IL-1*β* and IL-18 and better outcomes, which was mediated by the inhibition of NLRP3 inflammasome [12].

### 2.2. Glibenclamide and Severe Acute Pancreatitis (SAP)

Acute pancreatitis (AP), characterized by parenchymal and peripancreatic fat necrosis, often displays a mild and self-limiting inflammatory process and has a good prognosis. However, excessive inflammatory reactions result in severe acute pancreatitis (SAP), with a high mortality and morbidity [13]. Although the pathogenesis of SAP has not yet been clarified, inflammatory mediators such as IL-1*β*, IL-6, IL-8, IL-10, tumor necrosis factor-alpha (TNF)-*α*, platelet-activating factor, and monocyte chemotactic protein-1 (MCP-1) are considered vital for SAP development [38]. In vitro, glibenclamide significantly reduced IL-1*β* release in lipopolysaccharide- (LPS-) induced peritoneal cells [13]. In a mouse model of cerulien-induced AP, York et al. showed that glibenclamide (500 mg/kg, intraperitoneal) dramatically reduced serum levels of IL-6, lipase, and amylase [13]. Furthermore, in NLRP3 knockout (Nlrp3−/−) mice, cerulein significantly provoked less pancreatic edema, leukocyte infiltration, and acinar cell apoptosis compared to wild-type mice [39], which indicates the importance of the NLRP3 inflammasome in SAP pathogenies.

### 2.3. Glibenclamide and Urinary System Diseases

Inflammation plays a significant role in the progression of chronic kidney disease, both in animal experimental models [40] and in patients [41]. Expression of the NLRP3 inflammasome is enhanced in chronic kidney diseases [42]. Inflammatory markers, including TNF-*α* and NLRP3, were highly expressed in the kidneys from adenine diet-treated rats. Glibenclamide (10 mg/kg daily, 8 weeks) significantly reduced expression of TNF-*α* and NLRP3 [14, 15].

Bladder inflammation (cystitis) is a common and critical problem in urology. Hughes et al. indicated that NLRP3 played an important role in the pathogenesis of cyclophosphamide-induced cystitis, while glibenclamide could effectively block the inflammatory response [16]. In a cyclophosphamide-induced rat model of cystitis, glibenclamide treatment decreased caspase-1 activity along with levels of IL-1*β* and IL-18 and subsequent bladder inflammation [16].

### 2.4. Glibenclamide and Melioidosis

Melioidosis, caused by the Gram-negative saprophytic bacillus *Burkholderia pseudomallei*, results in bacteremia, abscesses in many organ systems, pneumonia, and soft tissue infection [43, 44]. DM is an established risk factor for susceptibility to melioidosis [12, 45–47]. However, a cohort study reported that melioidosis patients with a preadmission diagnosis of diabetes treated with glibenclamide had decreased levels of TNF-*α*, IL-18-R1, and IL-8 and lower in-hospital mortality compared to patients without diabetes [12]. The same group confirmed their findings in a mouse diabetes model of streptozocin-induced melioidosis [22]. Glibenclamide-pretreated diabetic mice showed lower *Burkholderia pseudomallei* loads in the liver and spleen, reduced neutrophils and macrophages infiltration in lung, and decreased IL-1*β* levels in the bronchoalveolar lavage fluid and lungs compared to control mice [22]. Whether glibenclamide displays a similar protective role against other infections is not known and needs further investigation.

Sepsis has been defined as a systemic inflammatory response to infection [48]. The newest definition for septic shock is “a subset of sepsis in which underlying circulatory, cellular, and metabolic abnormalities are associated with a greater risk of mortality than sepsis alone” [49]. Contradictory results regarding glibenclamide's administration in sepsis and septic shock have been reported. In a dog endotoxemia model, Landry and Oliver showed that glibenclamide (0.15 mg/kg, intravenous) effectively restored normotension [29]. Similarly, other studies showed that glibenclamide restored normotension in pig and rat endotoxemia models [27, 28]. However, sepsis or septic shock was also reported to be a negative effect of glibenclamide in experimental models and in human studies [27–29, 50–52]. In a ceacal ligation and puncture septic shock rat model, the administration of glibenclamide failed to improve cardiovascular and inflammatory parameters and actually increased mortality [50]. Similarly, in patients with septic shock, glibenclamide failed to improve hemodynamics or reduce noradrenaline requirements [51, 52]: in the randomized, double-blind, placebo-controlled crossover study, Warrillow et al. [51] found that glibenclamide (20 mg, enterally) treatment in patients with septic shock had no effect on hemodynamic variables, norepinephrine levels, or lactate concentrations but significantly decreased blood glucose concentration and increased dextrose utilization; Morelli et al. [52] performed another prospective, randomized, double-blind pilot study in patients with septic shock and showed that parenteral glibenclamide administration decreased blood glucose concentrations in a dose-dependent manner but failed to improve hemodynamics or reduce norepinephrine requirements. Possible explanations for this discrepancy might be related to species, pathogen, dose, and administration route (enteral versus intravenous) differences, which deserve further studies.

The proposed mechanisms of glibenclamide treatment of septic shock are reviewed as follows: first, glibenclamide could directly inhibit K_ATP_ channels in vascular smooth muscle (VSM). It is reported that K_ATP_ channels, especially Sur2B-Kir6.1 type, are abundantly expressed in vascular smooth muscle cells and have been suggested to regulate vascular tone in the presence of shock states and systemic inflammation [6, 53]. Under normal resting conditions, the K_ATP_ channel is closed, however, under increased tissue metabolism or tissue hypoxia conditions, and these channels are activated and opened [54]. A recent study by Shi and colleagues indicated that lipopolysaccharide upregulates expression of the K_ATP_ channel, especially the Kir6.1 and Sur2B subunits in smooth muscle cells dissociated from the mouse aorta [55]. Activation of K_ATP_ channels results in efflux of K^+^ and hyperpolarization of the cell membrane, which reduces Ca^2+^ influx and subsequently blocks constriction of vascular smooth muscle cells.

Second, the protective effect of glibenclamide might be associated with anti-inflammation, as it effectively reduced the lipopolysaccharide-induced release of mediators like IL-1*β* and TNF-*α* in vitro [56]. Many studies have shown that lipopolysaccharide-induced IL-1*β* production was NLRP3 inflammasome activation dependent [15, 57], while this activation could be inhibited by glibenclamide [58].

### 2.5. Glibenclamide and Cardiovascular Diseases

Atherosclerosis is the primary cause of ischemic heart diseases, and macrophages play a critical role in the pathogenesis of atherosclerosis and the formation of vulnerable plaque. The K_ATP_ subunits, especially Sur2A and Kir6.2, were upregulated on macrophages in vulnerable plaques, accompanied by the overproduction of TNF-*α* [17]. In vitro, glibenclamide (10 *μ*M) directly inhibited the lipopolysaccharide-induced TNF-*α* production by RAW264.7 cells [17]. In a mouse model of atherosclerosis, intragastric administration of glibenclamide at 2.5 mg/kg inhibited the formation/development of vulnerable plaque, diminished the vulnerability index (VI), and decreased the macrophage content in the plaque [17]. Furthermore, in a streptozocin-induced diabetic endotoxemia mouse model, pretreatment with glibenclamide (5 mg/kg i.p, 14 days) significantly attenuated myocardial injury via decreased production of IL-*β* and TNF-*α*, infiltration of macrophages, and apoptosis of cardiomycytes [59]. In vitro, glibenclamide pretreatment significantly decreased peritoneal macrophage IL-*β* expression but had no effect on TNF-*α* expression. Additionally, glibenclamide inhibited NLRP3 and caspase-1 expression [59].

### 2.6. Glibenclamide and Central Nervous System Diseases

The inflammatory response, especially sterile inflammation, is involved in the pathogenesis of CNS diseases, such as multiple sclerosis (MS) [21], progression of neurogenesis [20], and brain injury after subarachnoid hemorrhage (SAH) [18, 19]. Sur1-regulated channels, including K_ATP_ (Sur1-Kir6.2) channels and Sur1-Trpm4 channels, are expressed in neurons, astrocytes, oligodendrocytes, endothelial cells, and microglial cells [7, 8, 60]. Glibenclamide's inhibition of microglial K_ATP_ (Sur1-Kir6.2) channels and Sur1-Trpm4 channels significantly ameliorates neuroinflammation and improves neurological function.

Neural precursor cells are important in the progression of neurogenesis after brain injury. The K_ATP_ channels are expressed in microglial cells, where they are activated by cerebral ischemia and inflammatory stimuli [61, 62]. Ortega et al. reported that in lipopolysaccharide plus IFN-*γ*-stimulated microglial cells, glibenclamide treatment restored neural precursor cells activity by blocking K_ATP_ channels, inhibiting the microglia-induced suppression of neural precursor cell production, decreasing proinflammatory cytokines TNF-*α* and IL-6, and increasing MCP-1 [20]. Glibenclamide treatment enhanced MCP-1production, which was partially associated with enhanced neural precursor cell differentiation [63, 64].

Multiple sclerosis is an autoimmune disease characterized by chronic inflammation, demyelination, and neurodegeneration of the CNS that causes neurological disability [65, 66]. Using an experimental of autoimmune encephalomyelitis induced by MOG35–55 peptides in wild-type mice, Ortega et al. demonstrated that Sur1-Trpm4 channels were upregulated mainly on astrocytes [20]. The lumbar spinal cords of wild-type/experimental autoimmune encephalomyelitis mice treated with glibenclamide showed significantly less inflammatory lesions in leukocytes and T cells and less proinflammatory cytokines, including TNF-*α*, IFN-*γ*, and IL-17, which correlated with better preservation of myelin, axons, and mature and precursor oligodendrocytes [20].

SAH is a major contributor to hemorrhage stroke [67]. The Sur1-Trpm4 channel is upregulated in cortical tissues in a rat model of SAH, induced either by puncture of the internal carotid arteries [18] or by blood injection into the entorhinal cortex [19]. Glibenclamide treatment significantly reduced expression of proinflammatory cytokines TNF-*α* and nuclear factor-*κ*B (NF-*κ*B), decreased permeability and neuroinflammation, and ameliorated impaired spatial learning memory by blocking the Sur1-Trpm4 channel [19].

A recent study performed by Kurland et al. suggested that glibenclamide inhibition of lipopolysaccharide-treated microglial Sur1-Trpm4 channels downregulated inducible nitric oxide synthase (iNOS) transcription and thus reduced the formation of peroxynitrite radicals, which partly attributed to proinflammatory and harmful effects of activated microglia in the central nervous system [60].

### 2.7. Glibenclamdie and Ischemia-Reperfusion Injury

Ischemia-reperfusion (IR) injury usually results in the production of inflammatory mediators and reactive oxygen species induced by reperfusion. IR induces activation of K_ATP_ channels and cytokine production, such as IL-1*β*, IL-6, IL-17, and TNF-*α* [68–70]. Glibenclamide reduced the production of inflammatory mediators via inhibiting Sur1 and the regulatory subunit of the K_ATP_ channel in the ischemic tissue [61]. Recruitment and activation of leukocytes and subsequent release of inflammatory mediators have been proposed to explain the inflammation-induced reperfusion-associated injury [71]. Glibenclamide was suggested to inhibit neutrophil migration during the acute inflammatory response via blocking K_ATP_ channels [30]. The role of glibenclamide in different IR models was investigated, including the CNS [8, 23, 72], kidney [25], intestine [24], and testis [26].

The brain is very sensitive to ischemia; 5 minutes of ischemia can lead to irreversible neuronal cell death [23]. A review by Sun et al. indicated that K_ATP_ channel activation was important in the pathway of IR injury in the brain and was a promising target for protecting neurovascular function in stroke [70]. Another two studies performed by Simard et al. showed that glibenclamide treatment significantly reduced cerebral edema, infarct volume, and mortality in brain IR [8, 72]. Glibenclamide has been shown to ameliorate inflammatory mediators in the hippocampus in a rat IR model induced by occlusion of the bilateral carotid artery for 15 minutes followed by reperfusion for 60 minutes [23]. Glibenclamide reduced neutrophil infiltration, as well as TNF-*α* and prostaglandine E2 (PGE2), and boosted anti-inflammatory cytokine IL-10 expression [23].

Renal IR injury occurs in many clinical situations like organ transplantation, severe shock, and trauma. Both Kir6.2 and Sur1 are expressed in the normal renal epithelial cells [73] and upregulated in kidney IR [74]. In parallel with the increased expression of Kir and Sur, massive proinflammatory cytokines like IL-6, IL-17, and TNF-*α* increased [73]. Glibenclamide has generated contradictory results in kidney IR. Pompermayer et al. demonstrated that glibenclamide (20 mg/kg) was associated with amelioration of renal dysfunction in rat IR [25], characterized by decreased vascular permeability, neutrophil accumulation, NF-*κ*B translocation, and cytokine production like TNF-*α* [25]. In contrast, Zhang et al. showed that glibenclamide enhanced IR injury in renal epithelial function as demonstrated by altered histology in the kidneys of newborn rats [73]. Possible explanations for these contradictory findings could be related to age of the animals, since age influences calcium-activated potassium channels and voltage-activated potassium channel expression [75].

Increased vascular permeability, accumulation of neutrophils, as well as elevation of IL-1*β*, IL-6, and TNF-*α* was observed in a rat intestinal IR model. Histology showed marked intestinal destruction and inflammation, characterized by a striking loss of villi and crypts, and submucosal, hyperemia, and tissue edema [24]. Glibenclamide treatment significantly decreased intestinal IR injury in dose-dependent manner [24].

Testicular torsion, recognized as a urological emergency, can lead to testicular necrosis/apoptosis and decreased fertility in males, infants, and adolescents [76]. Reactive oxygen species produced in IR was responsible for the necrosis/apoptosis cascade [77]. Shimizu et al. showed that glibenclamide ameliorated the IR injury as demonstrated by a significant decrease in malondialdehyde concentration, myeloperoxidase activity, histological injury scores, and the number of TUNEL-positive germinal cells [26]. The binding of glibenclamide at the Sur1 subunit in the spermatogenic cells is a suggested mechanism since K_ATP_ channels are expressed on the sarcolemma, inner mitochondrial membrane, and nuclear membrane [7, 78, 79]. The role of glibenclamide in these inflammation-associated injury and disorders are summarized in Table 1.

## 3. Mechanisms of Glibenclamide Underlying Its Anti-Inflammatory Role

**Figure 1:**
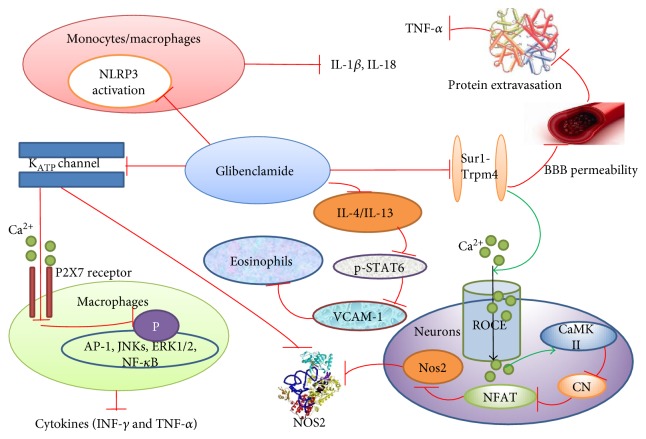
Anti-inflammatory mechanisms of glibenclamide. Glibenclamide blocks KATP channel to prevent K^+^ efflux and changes the membrane potential, which inhibits P2X7 receptor-mediated Ca^2+^ influx, resulting in reducing inflammatory cytokines. Glibenclamide blocks the Sur1-Trpm4 channel to decrease the BBB permeability, which reduces extravasated protein-induced production of TNF-*α*. Glibenclamide inhibition of Sur1-Trpm4 channel increases ROCE-mediated Ca^2+^ influx, which activates CaMKII and then inhibits CN/NFAT/Nos2 signaling. Glibenclamide can inhibit NLRP3 inflammasome-mediated production of IL-1*β* and reduce IL-13/4, which blocks the migration of eosinophils. The inhibitory effect is indicated by red lines with bar ends; the excitatory effect is indicated by green lines with arrow ends. ROCE: receptor-operated Ca^2+^ entry channel; CaMKII: Ca^2+^/calmodulin protein kinase II; CN: calcineurin; NFAT: nuclear of activated T cells; Nos2: inducible nitric oxide synthase gene; AP-1: activator protein-1; ERKs: extracellular signal-regulated kinases; JNKs: Jun N-terminal kinases; BBB: blood-brain barrier; p-STAT6: phosphorylated signal transducer and activator of transcription 6; VCAM-1: vascular cell adhesion molecular 1.

Glibenclamide exerts an anti-inflammatory effect involving inhibition of the NLRP3 inflammasome, decreased production of proinflammatory cytokines, reduced recruitment and migration of inflammatory cells, and production of nitric oxide (Figure 1).

### 3.1. Glibenclamide Might Inhibit the Activation of NLRP3 Inflammasome

In 2002, Martinon and coworkers introduced the term “inflammasomes” to describe a high-molecular-weight complex present in the cytosol of stimulated immune cells that mediates activation of inflammatory caspases [80]. The NLRP3 inflammasome (known as cryopyrin), like a number of NOD-like receptors (NLR), consists of the NLRP3 scaffold, the apoptosis-associated speck-like protein ASC/PYCARD adaptor and caspase-1, which is expressed predominantly in circulating monocytes and tissue macrophages [44]. It can be triggered by a plethora of pathogen-associated molecular patterns (PAMPs) and danger-associated molecular patterns (DAMPs) and leads to caspase-1 activation and cytokine secretion, including IL-18 and IL-1*β*, which are associated with tissue damage and chronic inflammation. Inappropriate activation of NLRP3 inflammasome has been linked to the pathophysiologic processes of many inflammatory diseases, such as gout, pseudogout, silicosis, and asbestosis [81–84]. Glibenclamide inhibited NLRP3 inflammasome activation and subsequently protected against organ inflammation and tissue damage either in a cerulean-induced obese mouse model of SAP, or in an adenine-diet rat model of chronic kidney disease, or in a cyclophosphamide-induced rat model of bladder inflammation, or in a lipopolysaccharide-induced myocardial injury in streptozocin-induced diabetic rats [13, 14, 16, 59]. Since potassium release and low-intracellular potassium concentration have been reported to trigger NLRP3 activation by all the stimuli both in mouse peritoneal macrophages and in human macrophages/monocytes [85, 86], inhibition of K_ATP_ channels that prevent depletion of cytosolic potassium might be a very plausible mechanism of glibenclamide-mediated suppression of the NLRP3 inflammasome [16]. In contrast, Lamkanfi et al. reported that K_ATP_ channels were dispensable for inhibition of the NLRP3 inflammasome [58]. As evidence showed that another sulfonylurea drug glipizide also inhibited Sur1 containing K_ATP_ channels but failed to inhibit NLRP3 inflammasome activation. Glibenclamide abolished inflammasome activation in macrophages lacking K_ATP_ channel subunits as well [58]. Although the exact mechanisms remain unknown, glibenclamide could inhibit NLRP3 inflammasome activation via upstream inhibition of the inflammasome and downstream blockade of the P2X7 receptor, which then reduces NLRP3 inflammasome-mediated capase-1 activation and inhibits secretion of the mature form of the IL-1*β* protein [58].

### 3.2. Glibenclamide Inhibits Production of Non-NLRP3 Inflammasome-Mediated Proinflammatory Cytokines

Excessive production of proinflammatory cytokines and recruitment of neutrophils are involved in the pathophysiology of tissue damage in inflammatory disease [30, 87]. In addition to IL-1*β*, glibenclamide also reduced NLRP3 noninflammasome-mediated proinflammatory cytokines, including TNF-*α* and INF-*γ*. Glibenclamide reduced lipopolysaccharide-induced TNF-*α* and INF-*γ* release and the corresponding mRNAs in monocytes by inhibiting the ATP/P2X7 receptor/calcium/AP-1 signaling [56], which was responsible for its protective role in lipopolysaccharide-induced sepsis shock [27–29]. Since cytosolic calcium was presumed to bind and activate transcription factors AP-1, which mediated the transcription of proinflammatory mediators in monocytes [88], the investigators ascribed the inhibitory potency of glibenclamide to its reduction of P2X7 receptor-induced calcium transients as well as the hypoxia-induced calcium elevations in monocytes [56]. ATP, released by injured erythrocytes, could activate P2X7 receptors on monocytes [89], which would provide a pore for calcium influx [56, 90]. P2X7 receptor activation depended on membrane potential, which could be influenced by Kir6.2 subunits of K_ATP_ channels expressed on monocytes [91]. Glibenclamide's effect on calcium was indirectly mediated by a change in membrane potential through inhibition of the Kir6.2 subunits [56].

Consistently, another study demonstrated that glibenclamide decreased TNF-*α* transcription by inhibiting the K_ATP_ channel as well. In the monocyte/macrophage RAW264.7 cell line, lipopolysaccharide significantly increased TNF-*α* transcript levels and robustly enhanced expression of K_ATP_ subunits, Sur1, Sur2A, Kir6.1, and Kir6.2 [17], which were depressed by glibenclamide. This study also provided strong evidence that the K_ATP_ channel was located upstream of NF-*κ*B as well as mitogen-activated protein kinases (MAPKS) in lipopolysaccharide-TLR (toll-like receptors) signaling. By inhibiting the K_ATP_ channel, glibenclamide suppressed phosphorylation of NF-*κ*B, extracellular signal-regulated kinases (ERK) 1/2 and Jun N-terminal kinases (JNKs) in RAW264.7 cells and therefore reduced the release of TNF-*α*, which rescued the progression of atherosclerosis in mice [17].

In both rat and human SAH models, glibenclamide could reduce the release of TNF-*α* and NF-*κ*B via inhibition Sur1-Trpm4 channel [18, 19]. The upregulation of Sur1-Trpm4 channel in SAH model resulted in increased blood-brain barrier permeability [18] and protein extravasation, which was related to the expression of TNF-*α* and NF-*κ*B and promotes neuroinflammation [92]. Glibenclamide treatment could significantly reduce the extravasation of serum proteins by blocking Sur1-Trpm4 channel and subsequently reduce TNF-*α* and NF-*κ*B expression.

### 3.3. Glibenclamide Inhibits Migration of Neutrophils and Eosinophils

Neutrophils are the main components of the innate immune system. Accumulation of neutrophils has been related to tissue damage due to their overwhelming release of cytotoxic and proinflammatory mediators, such as arachidonic acid metabolites, cytokines, superoxide anions, and nitric oxide [93] in infectious and ischemic diseases [23–25, 30]. Neutrophils have a high permeability to potassium, and their efflux occurs mainly through potassium channels [30]. Da Silva-Santos et al. showed that glibenclamide could effectively inhibit migration of neutrophils by blocking K_ATP_ channels [30], thereby reducing neutrophil accumulation and preventing further organ damage in ischemic brain, intestines, kidney, and lungs in a rat melioidosis model [22–25]. Additionally, glibenclamide's inhibitory effect on neutrophil migration is somewhat indirectly mediated by its inhibition of NLRP3 inflammasome assembly. NLRP3 inflammasome is the key for IL-1*β* maturation, and IL-1*β* upregulates the expression of endothelial intercellular adhesion molecules essential for the recruitment of neutrophils to the area of inflammation [94–96].

Eosinophils are potent effector cells implicated in allergic inflammation and helminth infection, predominantly involved in the pathogenesis of allergic asthma, eosinophilic bronchitis, and eosinophilic colitis. Cui et al. showed that glibenclamide could effectively inhibit eosinophil infiltration and reduced airway inflammation in mouse model of asthma [11]. Eosinophil recruitments are dependent on specific eosinophil chemokines like eotaxins and endothelial adhesion molecules like VCAM-1 [97, 98]. The production of eotaxins and VCAM-1 is regulated by IL-4 and IL-13. These cytokines promote the development of Th2 lymphocytes and activating structural cells like bronchial fibroblasts and respiratory epithelial cells to produce eotaxins and promote the expression of VCAM-1 in STAT6-dependent manner. Glibenclamide has been reported to reduce the production of Th2-associated cytokines, IL-4 and IL-13. Therefore, glibenclamide blockade of eosinophilic migration might due to the inhibition of IL-4/IL-13/phosphorylated STAT6/VCAM-1 signaling pathway. The mechanism underlying glibenclamide in the suppression of Th2 cytokines needs to be clarified in the future.

### 3.4. Glibenclamide Downregulates Generation of Reactive Oxygen Species

Excessive reactive oxygen species (ROS) triggers oxidative damage to biomolecules, resulting in the development of a variety of diseases, including ischemia-reperfusion injury, septic shock, and neuroinflammation [23, 26, 60]. There are various types of ROS, including oxygen-derived free radicals (superoxide, hydroxyl radicals, and nitric oxide) and nonradical oxygen derivatives of high reactivity (hydrogen peroxide, peroxynitrite, and hypochlorite) [99]. The generation of reactive oxygen species is dependent on specialized enzymes, such as NADPH-oxidase, myeloperoxidase, and nitric oxide synthase (NOS). Three isoforms of NOS have been identified: endothelial (eNOS), neuronal (nNOS) (both constitutively expressed), and inducible (iNOS) (produced primarily after stimulation by cytokines and endotoxin).

Shimizu et al. reported that glibenclamide ameliorated the rat testis IR by reducing the myeloperoxidase, and thus reducing the oxidative stress [26]. But the underlying mechanism of inhibition myeloperoxidase by glibenclamide is not clear. The authors suggested that this might be mediated by blockade membrane K_ATP_ channel, since the openers of membrane K_ATP_ channel do not reduced injury. In addition to myeloperoxidase, glibenclamide could inhibit NOS2 expression. Overproduction of nitric oxide due to NOS2 activation has been found in septic shock and is suggested to be one of the mechanisms underlying hypotension and vascular hyporeactivity to vasoconstrictors [100–102]. Wu et al. showed that glibenclamide inhibited NOS2 induction caused by LPS in cultured macrophages and in the anaesthetized rat [103]. This explains the beneficial effects of glibenclamide in endotoxin-induced shock. The inflammatory cytokine TNF-*α* has been reported to upregulate NOS2, thereby mediated a generalized production of the potent vasodilator, nitric oxide [104]. Since glibenclamide effectively reduced the production of TNF-*α* in septic shock [56], it might be possible that glibenclamide downregulates NOS2 by inhibiting TNF-*α*. This mechanism needs to be investigated.

Furthermore, a recent study indicated that glibenclamide reduced *Nos2* mRNA dose dependently in activated microglia by blocking Sur1-Trpm4 channels [60]. NOS2 expression in activated microglia is regulated by NFATc1 (a kind of NFAT, nuclear factor of activated T cells) [105], which is phosphorylated and sequenced in the cytoplasm. Nuclear translocation occurs following dephosphorylation of NFATc1 by the Ca^2+^-sensitive phosphatase, calcineurin (CN) [106]. Sur1-Trpm4 channel in microglia has been shown to be significantly upregulated by LPS stimulation in vivo and in vitro. The activation of Sur1-Trpm4 channels in activated microglia depolarizes the cell membrane, which decreases the inward driving force for Ca^2+^ [7]. While glibenclamide inhibition of Sur1-Trpm4 leads to increase in intracellular concentration of Ca^2+^ ([Ca^2+^]_i_), which causes the activation of CaMKII (Ca^2+^/calmodulin protein kinase II) [107]. Activated CaMKII inhibits the phosphatase activity of CN through phosphorylation [108], thus fails to dephosphorylate NFATc1 and inhibits the nuclear translocation [107, 108], which results in induction of *Nos2* mRNA induction. The nitrite production by NOS2 in activated microglia is attributable to neuroinflammation and injury in a growing list of CNS diseases. The beneficial effects of glibenclamide in multiple sclerosis, ischemic stroke, and hemorrhage stroke might be mediated by the inhibition of Sur1-Trpm4-[Ca^2+^]_i_-CN/NFAT-*Nos2* signaling. Glibenclamide could also inhibit oxidative stress by elevating activity of antioxidant enzyme, such as glutathione peroxidase, superoxide dismutase, and catalase [23, 109].

Hypoglycemia, the most common side effect of glibenclamide, can result in large releases of norepinephrine, epinephrine, and steroids, which can inhibit macrophage migration and production of TNF-*α* [110, 111]. Studies involved in our review showed that patients who were not pretreated had no obvious changes in blood glucose levels [10–12, 17–19, 22–26, 59]. This finding indicates that the anti-inflammatory effect of glibenclamide is independent of its hypoglycemic effect.

## 4. Conclusions

Glibenclamide exerts a protective role in inflammation-related injury and disorders. With the blockades of NLRP3 inflammasome/IL-1*β* signaling and non-NLRP3 mechanisms like Sur1-Trpm4/TNF-*α* signaling and Sur1-Trpm4/Nos2/ROS signaling, glibenclamide downregulates proinflammatory cytokines and reactive oxygen species and suppresses migration of inflammatory cells. Glibenclamide might be a promising agent for inflammation-associated disorders. Further investigations could focus on translating the anti-inflammatory potential of glibenclamide to clinical application in inflammatory diseases.

## Figures and Tables

**Table 1:** The anti-inflammatory effects of glibenclamide in previous studies.

Objects	Anti-inflammatory effects
Bronchopulmonary dysplasia model of mice [10]	Decreases in IL-1*β*, neutrophils, and macrophages in bronchoalveolar lavage fluid
Allergic asthma model of mice [11]	Inhibitions in airway hyperresponsiveness, airway inflammation, and Th2 cytokines
Severe acute pancreatitis model of mice [13]	Decreases in serum levels of IL-6, IL-1*β*, lipase, and amylase
Cystitis model of rats [16]	Decreases in bladder mucosa edema, neutrophils infiltration, and proinflammatory cytokines expression
Sepsis patients [12]	Attenuations of inflammatory responses and mortality
Sepsis model of mice [22]	Suppressions of inflammatory cells in the lung, bacterial dissemination, and IL-1*β* secretion
Atherosclerosis model of mice [17]	Diminishment of vulnerability index and decrease in macrophages infiltration in plaque
Lipopolysaccharide-treated RAW264.7 cells [17]	Inhibition of TNF-*α* expression
Endotoxemia model of mice [59]	Attenuations of myocardial injury and macrophages infiltration and IL-1*β* expression
Autoimmune encephalomyelitis model of mice [21]	Ameliorations of inflammatory cells and cytokines
Neural precursor cells treated with lipopolysaccharide and IFN-*γ* [20]	Restoration of lipopolysaccharide and IFN-*γ* induced decrease in neural precursor cell number
SAH model of rats [19]	Amelioration of brain-blood barrier permeability, decreased expression of proinflammatory cytokines like TNF-*α*, and suppression of neuron cell death
Brain IRI model of rats [23]	Amelioration of neutrophil infiltration, inhibitions in TNF-*α*, and PGE2 expressions, and upregulation of anti-inflammatory cytokine IL-10
Renal IRI model of rats [25]	Amelioration of vascular permeability and inhibitions in neutrophil accumulation and TNF-*α* production
Intestinal IRI model of rats [24]	Amelioration of vascular permeability, inhibition in neutrophil accumulation, and suppressive productions of cytokines like TNF-*α*, IL-1*β*, and IL-6
Testis IRI model of rats [26]	Downregulations of malondialdehyde concentration, myeloperoxide activity, and histological score

SAH: subarachnoid hemorrhage; IRI: ischemia-reperfusion-induced injury.
